# Are pictures worth a thousand sleep signals?

**DOI:** 10.1093/sleep/zsad268

**Published:** 2023-10-16

**Authors:** Steven Holfinger

**Affiliations:** Department of Internal Medicine, The Ohio State University Medical Center, Columbus, OH, USA

Expert epoch-by-epoch visual analysis of the electroencephalogram (EEG) using attended polysomnography (PSG) is the current gold standard for measuring sleep, despite being a time-consuming, and imperfect process. Automated analysis of PSG aims to improve scoring accuracy, reduce variability, and increase practice efficiency. However, this process is limited by the (1) variability of sensors and resolution of recorded signals used by different sleep laboratories, (2) differences in PSG analysis software and data formats, (3) challenges in assessing performance of automated analysis due to heterogeneity of training and testing datasets, and (4) real-world implementation problems related to integration into current systems, billing and coding requirements, and acceptance by practitioners [1].

In this issue of *SLEEP*, Shin et al. describe an image-based approach to automatically score sleep stages in a newly assembled, large dataset [2]. This new dataset includes 10 253 PSG studies from several Korean institutions and may be accessible to other investigators if permission is granted by the Korean government. Data were stored in European Data Format, annotated JavaScript Object Notation (JSON) files, and in their newly described image format.

The new image-based scoring algorithm applied deep learning methods to analyze images containing 11 PSG biosignals (three acceleration, four EEG, two electrooculogram (EOG), Chin Electromyograph (EMG), electrocardiogram (ECG), Flow, Thermistor, Thorax/abdominal movement, two snores, two leg EMG, two Oxygen saturation) in 30-second epochs. It was compared against a second deep learning algorithm trained on the same dataset, except using signal-based data rather than image-based data. The two had similar levels of overall sleep-scoring accuracy in the hold-out validation set, 82.9% versus 81.9% in the image-based versus signal-based models, respectively. The image-based model was also compared using F1 scores against the Sleep Heart Health Study (SHHS) dataset, which notably was missing two of the EEG channels [3]. F1 scores, an accuracy metric combining precision and recall, can be averaged across each predicted sleep stage to determine the Macro F1 score. The Macro F1 scores in the internal validation set were 82.9% and 80.9% for the image-based and signal-based methods, respectively, while the Macro F1 score in the SHHS using the imaged-based method reached 69.2%.

By standardizing each epoch of the PSG in a visually similar way to human analysis (the images look like screenshots taken during human scoring), this image-based approach has several strengths. Using a common image format theoretically should be capable of receiving inputs from all PSG formats, as the differences between hardware and software resolve when converted to the standardized image format. In practice, 88.5% of the prescreened PSG successfully converted into the image-based format and passed manual review [2]. Another benefit of the image-based analysis is the addition of Eigen-CAM to visually show the end user areas used by the model for classification. The black-box nature of automated analysis can lead to a delay in adoption and skepticism of unproven algorithms, and the use of the heat map to visually display areas of interest may mitigate some of these concerns.

From a high-level perspective, one must consider how these results are achieved despite a significant reduction in signal quality. The process of converting the signals to the image-based format effectively limited any analysis requiring precise data. For example, after resizing to 224 × 224, each ECG pixel represented 0.112 mV vertically, and 0.134 seconds horizontally. The Nyquist limit posits the maximum discernable frequency would be 3.7 Hz, which is significantly lower than what is required for heart rate variability (HRV) analysis. Reducing the ECG sampling rate to 50 Hz has been shown to produce unacceptable HRV results, so it can be assumed the image-based analysis does not utilize HRV [4]. Yet, the accuracy findings from the validation set near human levels of performance. This suggests that (1) relevant data needed to approximate human scoring is possible using only low-resolution images, (2) state-of-the-art machine-learning methods used for image analysis may be superior to alternative signal analysis methods, and (3) these results may only be true for patients similar to those in the training dataset, and outside validation is needed for widespread adoption. I suspect all are true here, as the ResNet101 model only was beneficial when used in the image-based dataset and there was a drop in accuracy when applied to the outside SHHS dataset.

As is the case for most automated scoring systems, this model would benefit from further investigation to better understand real-world performance. The reduction in performance seen in the SHHS dataset may have been due to the missing EEG signals or could be a sign of reduced inter-database generalization. However, there remains a larger question of using human-based, 30-second epoch scoring as the gold standard when training new models [1]. A recent meta-analysis estimated the interrater reliability between expert PSG scorers at a Cohen’s kappa of 0.76 for overall sleep staging agreement between individuals, when looking for exact agreement in staging between scorers [5]. A possible proposed solution shows the agreement is better if considering equivocal epochs, meaning epochs with stages agreed upon by some (but not a majority) of expert scorers when features of multiple sleep stages exist within the same epoch. Using this method to consider equivocal epochs demonstrates accuracy can be as high as 96%, compared to the typical results using a “majority-rules” approach at about 82% [6].

Another approach is to transcend human-imposed limitations on the dataset, such as the 30-second epoch and traditional stages, and directly correlate novel sleep measures to relevant health outcomes such as morbidity/mortality. Promising approaches include the EEG-derived odds-ratio product and cardiopulmonary coupling (CPC) to form continuous/alternative measures of sleep [7, 8]. As consumer and home sleep testing devices continue to improve, alternatives to manual scoring PSG will likely define the future of sleep medicine.
